# Significant suppression of radiation dermatitis in breast cancer patients using a topically applied adrenergic vasoconstrictor

**DOI:** 10.1186/s13014-017-0940-7

**Published:** 2017-12-22

**Authors:** James F. Cleary, Bethany M. Anderson, Jens C. Eickhoff, Deepak Khuntia, William E. Fahl

**Affiliations:** 1 Department of Medicine, University of Wisconsin, Madison, WI USA; 2 Department of Human Oncology, University of Wisconsin, Madison, WI USA; 3 Department of Biostatistics and Medical Informatics, University of Wisconsin, Madison, WI USA; 4 Varian Medical Systems, Palo Alto, CA USA; 5 Valley Medical Oncology, Pleasanton, CA USA; 6 Department of Oncology, Wisconsin Institutes of Medical Research, University of Wisconsin-Madison, 1111 Highland Avenue, Madison, WI USA

**Keywords:** ROS, Phase IIa clinical trial

## Abstract

**Background:**

Our previous studies showed that vasoconstrictor applied topically to rat skin minutes before irradiation completely prevented radiodermatitis. Here we report on a Phase IIa study of topically applied NG12-1 vasoconstrictor to prevent radiodermatitis in post-lumpectomy breast cancer patients who received at least 40 Gray to the whole breast using standard regimens.

**Methods:**

Patients had undergone surgery for Stage Ia, Ib, or IIa infiltrating ductal or lobular carcinoma of the breast or ductal carcinoma in situ. NG12-1 formulation was applied topically to the same 50-cm^2^ treatment site within the radiation field 20 min before each daily radiotherapy fraction.

**Results:**

Scores indicated significant reductions in radiodermatitis at the NG12-1 treatment site versus control areas in the same radiotherapy field. The mean dermatitis score for all subjects was 0.47 (SD 0.24) in the NG12-1-treated area versus 0.72 (SD 0.22) in the control area (*P* = 0.022). Analysis by two independent investigators indicated radiodermatitis reductions in 9 of the 9 patients with scorable radiodermatitis severity, and one patient with insufficient radiodermatitis to enable scoring. There were no serious adverse events from NG12-1 treatment.

**Conclusions:**

Thirty, daily, NG12-1 treatments, topically applied minutes before radiotherapy, were well tolerated and conferred statistically significant reductions in radiodermatitis severity (*P* = 0.022).

**Trial registration:**

NCT01263366; clinicaltrials.gov

## Background

Of the estimated 230,000 new breast cancers diagnosed in the US in 2013 [1] many were treated with lumpectomy followed by radiotherapy. Though linear accelerators produce photon beams that preferentially provide deep tissue penetration, a significant radiation dose is deposited within the skin. Acute skin radiation reactions typically appear 10-14 days from the start of radiotherapy and increase in severity through the end of treatment [2]. Radiodermatitis is the primary treatment-limiting acute side effect of adjuvant breast radiation and may cause treatment breaks and delays that have an adverse impact on tumor recurrence [3–6].

With an occasional exception [7], the prevention and treatment of radiodermatitis have been pursued for decades with little or no success. For example, there was no evidence for radiodermatitis prevention when topical methylprednisolone and dexpanthenol were studied in breast cancer patients undergoing fractionated radiotherapy [8]. Topical agents that have been evaluated include *Aloe vera*, Biafine, and steroidal and nonsteroidal anti-inflammatory agents, but none have been shown to be clinically effective [3, 9, 10].

The present clinical trial follows from our earlier observation that topically applied adrenergic vasoconstrictors conferred 100% radiodermatitis prevention in a rat model when applied to the skin a few minutes before irradiation [11]. In this strategy, the vasoconstrictor penetrates into the skin to constrict blood vessels about 1 mm below the skin surface; this causes local, transient hypoxia in the overlying 1 mm skin layer in which epidermal stem cells lie. The skin hypoxia reduces radiation-induced reactive oxygen species (ROS) formation and directly inhibits “fixation” of ROS-induced DNA damage; the hypoxia thus suppresses radiation-induced chemical and physical damage to epithelial stem cell DNA and reduces skin toxicity.

The delivery of an adrenergic vasoconstrictor through the stratum corneum to subcutaneous vasculature is accomplished using high concentrations of adrenergic vasoconstrictor in an aqueous alcohol-based topical delivery vehicle. The alcohol content decreases surface tension, enables drug “spread” on the skin, fluidizes lipids within the stratum corneum, and enables drug loading into the epidermis [12]. The alcohol also fluidizes sebum, which enables drug passage down hair follicle channels to the subcutaneous vasculature that forms a meshwork around the hair follicle stem cell bulbs [13].

The adrenergic vasoconstrictor NG12-1 formulation was chosen as the first vasoconstrictor to be tested in this Phase IIa proof of concept study. Mechanistically, the topically applied adrenergic drug: i) vasoconstricts subcutaneous vessels to produce transient skin hypoxia during the radiotherapy session; this also prevents systemic distribution of the topically applied vasoconstrictor, and ii) is rapidly metabolized by the catabolic enzymes catechol-O-methyl transferase, COMT, and monoamine oxidase, MAO, both abundantly present in skin [14]; this also makes the vasoconstriction transient.

In two previous Phase I safety studies (unpublished), we evaluated the safety of the NG12-1 formulation in i) normal volunteers and ii) cancer patients receiving palliative radiotherapy to the spine. In the first Phase I study, 18 healthy volunteers received daily topical NG12-1 treatments, 5 days each week for 6 weeks, to mimic a radiotherapy treatment schedule. Four increasing dose levels of NG12-1 were tested for both safety, as well as degree and duration of skin blanch. There were zero observations of blood pressure elevation, local skin necrosis, or any significant treatment-associated adverse events. Both the skin blanch degree and duration showed direct dependency on the topically applied NG12-1 dose. In the second Phase I safety study, 13 cancer patients were enrolled. All of the patients had spinal bone metastases that were irradiated with a modest radiation dose that historically rarely elicited radiodermatitis. Topical application of the NG12-1 formulation within the radiation field was well tolerated; there were no adverse events noted, either systemically or to the drug-treated, irradiated skin.

The present Phase IIa study of NG12-1 was a prospective, nonrandomized, open-label, safety and efficacy study in post-surgical breast cancer patients treated with radiation therapy. The topical NG12-1 formulation was applied on each radiotherapy day to the same “study drug application site” (SDAS), a 50-cm^2^ area within the chest-axilla radiation field, approximately 20 min prior to each radiation fraction. Both the SDAS and surrounding radiotherapy field were photographed and scored for radiodermatitis severity before, during and after the radiotherapy treatment course.

## Methods

This study (#NCT01263366; http://www.clinicaltrials.gov) was designed and conducted in accordance with the principles outlined in the Declaration of Helsinki and within the guidelines of Good Clinical Practices, and approved by the University of Wisconsin Health Sciences IRB. Informed consent was obtained from all subjects who participated in the study.

Eligible subjects were women, age 18+, with Stage Ia (T1, N0, M0), Ib (T0 or 1, N1mic, M0) or IIa (*T* < 3 cm, N0, M0) infiltrating ductal or lobular carcinoma of the breast or ductal carcinoma in situ, scheduled to receive fractionated radiotherapy following breast-conserving surgery. The minimum permitted dose was 40 Gray (Gy) as 15-28 divided fractions to the breast and axilla. An additional boost to the lumpectomy area was permitted.

Exclusions included rashes, ulcerations and unhealed surgical wounds in the SDAS; generalized skin or connective tissue disorders; active untreated cardiac disease, uncontrolled hypertension or clinically significant abnormal ECG; use of MAO inhibitors or triptyline or imipramine antidepressants; lymphovascular space invasion, dermal lymphatic invasion on pathology, or inflammatory breast cancer; proximity of the tumor to the overlying skin (depth < 5 mm within 2 cm of the SDAS); concurrent chemotherapy other than Herceptin; and previous radiation to the breast.

The radiation field was defined and the boundary of the 50-cm^2^ SDAS was marked with at least 2 cm inside the upper and lateral edge of the radiation field. At each treatment and follow-up visit, the study nurse assessed the SDAS and surrounding radiation field, measured blood pressure and pulse, and applied the topical NG12-1 formulation approximately 20 min prior to radiotherapy. Digital photographs were taken at least once each treatment week and at follow-up visits. The radiation oncologist performed a visual assessment once each week and at each follow-up visit.

The NG12-1 drug and formulation, provided by Suzhou NG Biomedicine Ltd., is described in NCT01263366 at http://clinicaltrials.gov. A sponge applicator was used to spread 3.0 mL of the topical formulation onto skin within the marked boundaries of the SDAS.

The time window for study drug application was 10-30 min prior to radiation therapy, preferably 20 min; this corresponded to the previously determined time to maximum skin blanch intensity.

Toxicities were categorized using NCI CTCAE version 4.0 criteria. Each toxicity event was assigned an attribution: unrelated, unlikely, possibly, probably, or definitely topical drug-treatment related. Toxicities were summarized in tabular format. All subjects who received at least one application of topical NG12-1were evaluable for toxicity.

Efficacy was assessed by comparing the highest radiodermatitis grade (NCI CTCAE version 4.0 criteria for Rash) within the SDAS to the highest grade in the untreated area surrounding the SDAS. Radiodermatitis scores were summarized in terms of means and standard deviations. The nonparametric Wilcoxon Signed Rank test was used to compare the radiodermatitis scores between the NG12-1-treated versus untreated sites. A positive efficacy response was defined as a reduction in the maximum severity of dermatitis at the study drug treatment site relative to the adjacent area by one grade level or more (NCI-CTCAE Version 4.0 criteria for Rash). Efficacy responses were summarized in tabular format. The 95% confidence interval for the overall efficacy response rate was constructed using the exact binomial distribution. All *P* values were 2-sided and were considered significant at <0.05. Statistical analyses were performed with SAS software (SAS Institute, Inc., Cary, NC), version 9.2.

After nine subjects completed treatment, a board-certified radiation oncologist performed a protocol-specified interim analysis, using digital images obtained from the principal investigator. The analysis included a descriptive summary for each subject, and an efficacy score for reduction in the severity of radiodermatitis according to the following scale:

+++ Strong evidence.

++ Good evidence.

+ Some evidence.

0 No evidence.

The tenth subject enrolled and was treated subsequent to the interim analysis. A descriptive summary and score were prepared at a later date.

## Results

Eleven subjects enrolled, but only ten were treated with topical NG12-1. All subjects were white non-Hispanic females, age 47 to 68 years (Table 1, Study Population Baseline Characteristics). One subject presented with hypertension on the first two radiotherapy days; she was removed from the study and was ineligible for safety and efficacy assessment.

**Table 1:** Study population baseline characteristics

Age (yrs)	Mean (SD)	59 (6.6)
	Median (Range)	58 (47-68)
Gender	Female	11 (100%)
Race	White	11 (100%)
Ethnicity	White	11 (100%)
Pathology (TNM Stage)	Tis	4 (36.4%)
	T1a	1 (9.1%)
	T1b	1 (9.1%)
	T1c	5 (45.5%)
	N0	9 (81.8%)
	NX	2 (18.2%)
	M0	4 (36.4%)
	MX	7 (63.6%)
	Tumor Size (cm), Mean (SD)	1.25 (0.60)
	Median (Range)	1.4 (0.2-2.2)
Eligible for Safety and Efficacy	Yes (received topical NG12-1)	10
	No (did not receive topical NG12-1)	1
Surgical Procedure	Lumpectomy	9
	Partial Mastectomy	2
Radiation Regimen	Standard Regimen without Boost	2
	Standard Regimen with Boost	4
	Hypofractionated Regimen without Boost	1
	Hypofractionated Regimen with Boost	3
	Unknown (did not receive topical norepinephrine)	1

### Safety evaluations

There were no serious adverse events, patient withdrawals, or premature terminations. None of the non-serious adverse events were considered probably or definitely related to study drug (Table 2, Adverse Events by System Organ Class). There was no evidence for any blood pressure elevation or tachycardia related to topical vasoconstrictor application.

**Table 2:** Adverse Events: Possibly or Unlikely Related to Treatment^a^

Description	Subject	Grade	Attribution	Outcome
System Organ Class: Injury, poisoning and procedural complications				
Transient, warm/burning feeling	2002	1	Possibly	Recovered/Resolved without sequelae
Tingling to right (treated) breast	2005	1	Unlikely	Recovered/Resolved without sequelae
Breast edema (treated breast)^b^	2005	1	Unlikely	Recovered/Resolved without sequelae
System Organ Class: Nervous system disorders				
Dizziness	2006	1	Unlikely	Recovered/Resolved without sequelae
System Organ Class: Reproductive system and breast disorders				
Increased frequency of hot flashes	2004	1	Unlikely	Recovering/Resolving

^a^Toxicities were graded using NCI CTCAE version 4.0 criteria. No subject experienced an adverse event considered definitely or probably related to NG12-1 treatment

^b^Edema is a known manifestation of radiodermatitis

### Efficacy evaluations

**Fig. 1:**
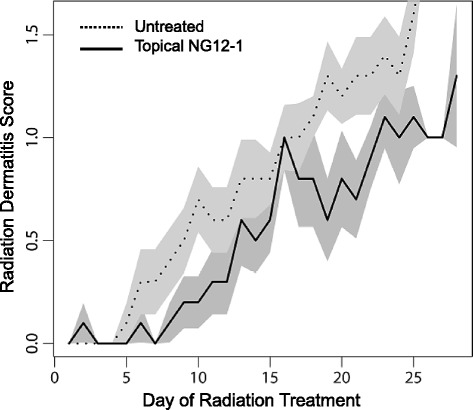
Radiodermatitis scores for topical NG12-1-treated versus control sites, plotted versus the day of radiation treatment

Radiodermatitis severity scoring was based on toxicity grades assigned by the study principal investigator to the SDAS and the adjacent untreated control site of every patient on each of the ~ 25-30 daily treatment visits in a standard 50 Gy radiotherapy regimen. The combined average radiodermatis severity scores for Patients 1-9 on every treatment day, for example the nine scores for Patients 1-9 on day 10 of their radiotherapy regimen, were calculated for both the NG12-1 (i.e., SDAS) and the vehicle control treatment areas, and these average scores were then plotted (Fig. 1). Comparison of the averaged scores for SDAS and control skin in Patients 1-9, on every day of the 30 day radiotherapy regimen, is shown in Fig. 1. The combined radiation dermatitis score for all subjects in the NG12-1-treated area was 0.47 (SD 0.24) versus 0.72 (SD 0.22) in the untreated control area, a statistically significant difference (*P* = 0.022). The overall drug efficacy response rate was 100% (95% CI: 69% - 100%).

Nine of the 10 subjects were included in the clinical protocol-specified Interim Analysis (Table 3, Interim Analysis). Three subjects (2003, 2004 and 2005) were scored +++ (strong evidence for a reduction in the severity of radiodermatitis), two (2000 and 2001) were scored ++ (good evidence), three were scored + (some evidence) and one was scored 0 (no evidence). The tenth subject (2009, 50 Gy in 25 fractions) was not included in the Interim Analysis, but was scored (+) in the final analysis by the same reviewer.

**Table 3:** Interim Analysis

Subject	Regimen	Reviewer’s Assessment	Score
2000	50 Gy/25 Fx Boost 10 Gy/5 Fx	Grade 2 dermatitis and Grade 1 folliculitis on treatment day 25. On boost day 5, the dermatitis in the axilla had improved. The SDAS showed an area of protection.	++
2001	45 Gy/25 Fx, Boost 16 Gy/8 Fx	On boost day 5, Grade 1 dermatitis in the posterior axilla with Grade 2 dry desquamation in the anterior axilla. At Follow-up, the SDAS showed a subtle decrease in dermatitis relative to the rest of the axilla. Treatment benefit evident late.	++
2002	50.4 Gy/28 Fx, Boost 10 Gy/5 Fx	On treatment day 25 there was an area of Grade 2 dry desquamation just outside of the SDAS. The drug may have prevented desquamation in the SDAS	+
2003	50.5 Gy/25 Fx	Grade 1 dermatitis with an area of Grade 2 dry desquamation in the axilla, adjacent to the SDAS. The axilla had less dermatitis than expected; the SDAS was Grade 0.	+++
2004	50 Gy/25 Fx, Boost 10 Gy/5 Fx	SDAS had a response that was evident on treatment day 23 and obvious on boost day 5, when there was Grade 0 dermatitis in the SDAS vs Grade 1-2 elsewhere.	+++
2005	42.66 Gy/16 Fx, Boost, 8 Gy/4 Fx	Grade 1 dermatitis. On boost day 4, an area in the axilla looked dusky, as if there was about to be a skin breakdown. At week 4 follow-up, the SDAS seemed to have improved more than other areas	+
2006	50.4 Gy/28 Fx, Boost 10 Gy/5	At week 1 follow-up, Grade 1 dermatitis in the axilla and Grade 2 dermatitis above the nipple. Obvious reduction in the SDAS, subtle on boost day 2 and obvious at week 1 follow-up, when there was Grade 0 dermatitis and a clear response relative to the rest of the breast.	+++
2007	42.66 Gy/16 Fx	Grade 1 radiodermatitis and folliculitis. No changes in the SDAS on day 15, but a subtle improvement at week 1 follow-up.	+
2008	42.56 Gy/16 Fx, Boost 8 Gy/4 Fx	Very mild grade 1 radiodermatitis on the breast and in the axilla.	0

**Fig. 2:**
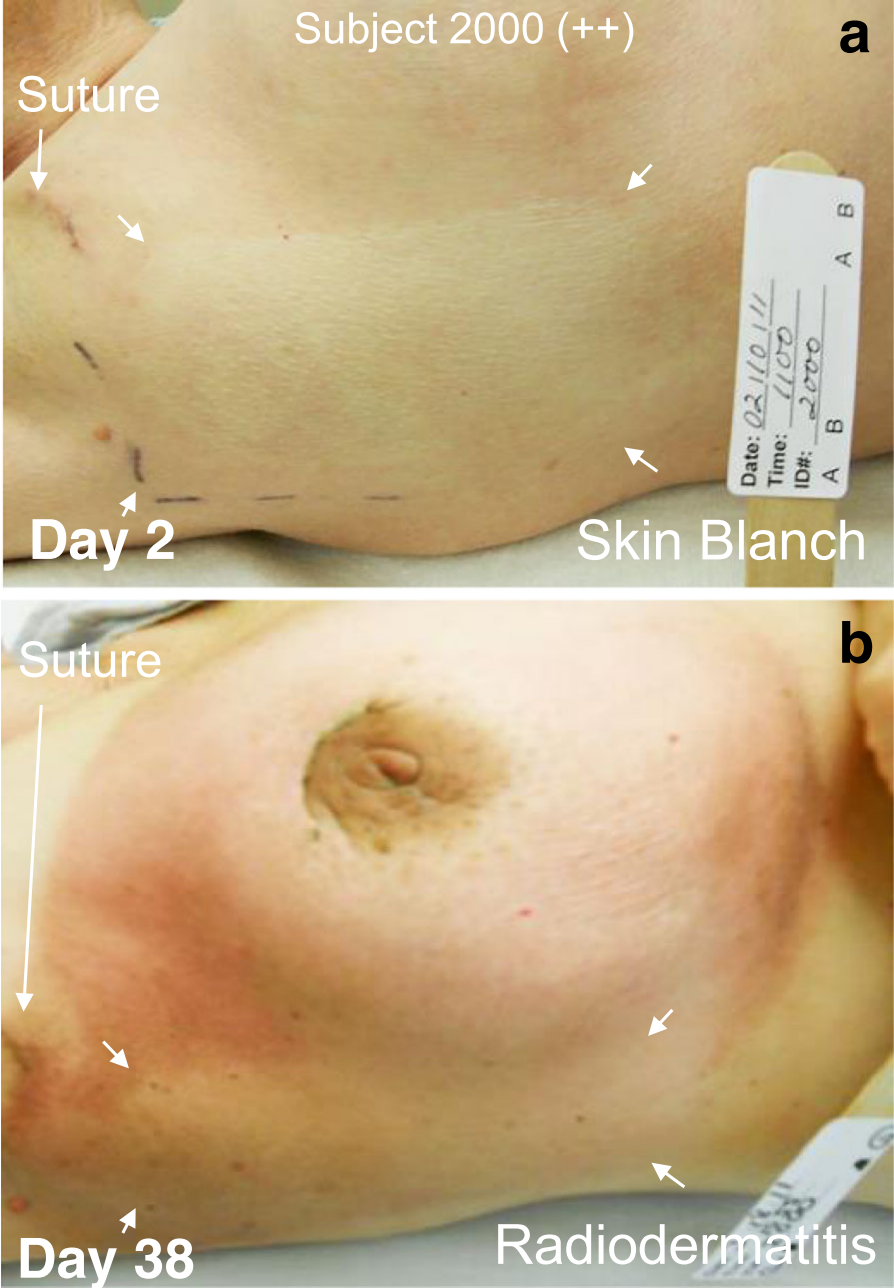
Subject 2000. A, NG12-1 treatment area (arrows) showing skin blanch 20 min after topical NG12-1 application. B, NG12-1 treatment area (arrows) showing reduced radiodermatis on Day 38. The interim analysis score was ++

**Fig. 3:**
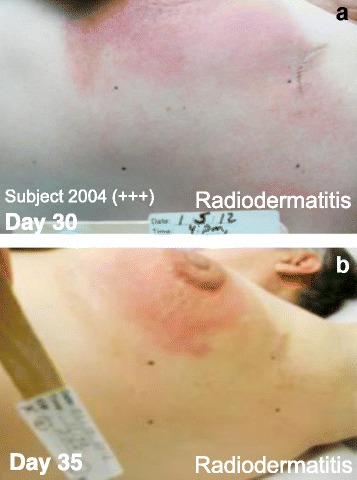
Subject 2004. NG12-1 treatment area (ink marks) showing reduced radiodermatis on Day 30 (Panel **a**) and Day 35 (Panel **b**). The interim analysis score was +++

**Fig. 4:**
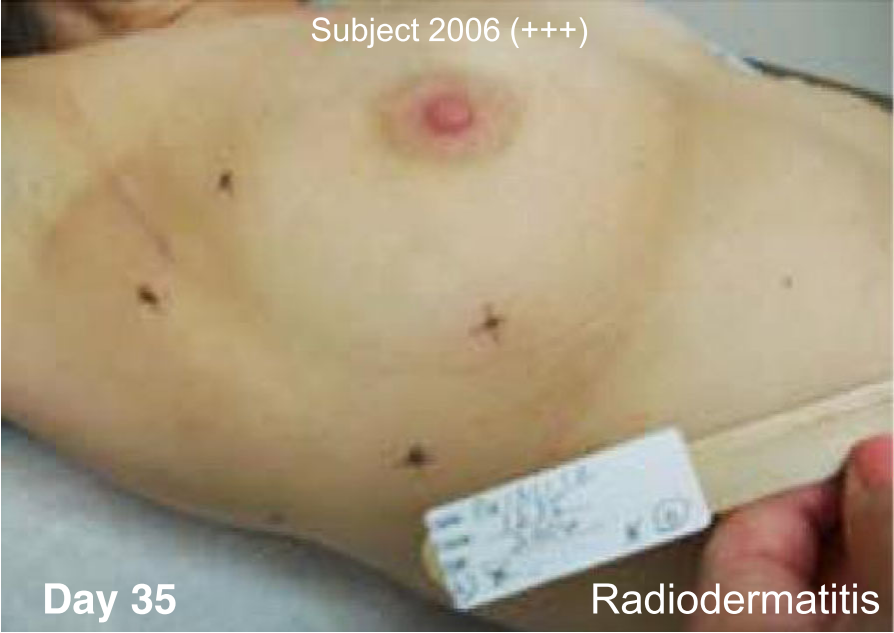
Subject 2006. NG12-1 treatment area (ink marks) showing reduced radiodermatis on Day 35. The interim analysis score was +++

**Fig. 5:**
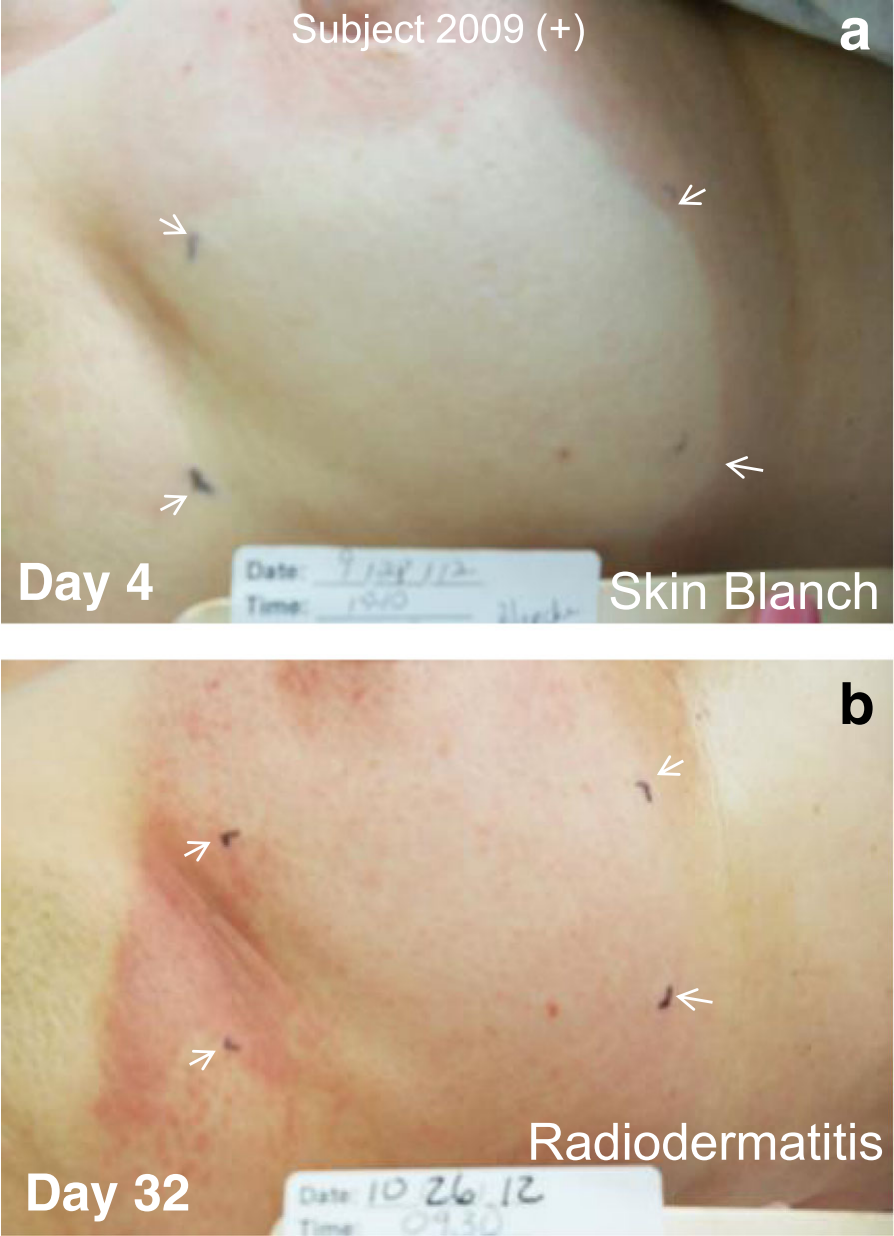
Subject 2009. A, NG12-1 treatment area (arrows) showing skin blanch 20 min after topical NG12-1 application on Day 4. B, NG12-1 treatment area (arrows) showing reduced radiodermatis on Day 32. The final analysis score was +

Images illustrating the reduction in radiodermatis severity within the NG12-1- treated SDAS for Subject 2000 (Fig. 2), Subject 2004 (Fig. 3), Subject 2006 (Fig. 4) and Subject 2009 (Fig. 5) are shown. Figs. 2a and 5a also show that the area of NG12-1-induced skin blanch directly overlaid the area in which radiodermatitis was subsequently significantly reduced.

## Discussion

Current best clinical practices for the prevention and treatment of acute radiation dermatitis are limited to agents that minimize discomfort, promote healing, or prevent infection [3, 10]. No interventions to date have been shown to be clinically effective, and none address the core problem of preventing or minimizing acute radiation damage to the skin. To our knowledge, this is the first report of a topical or systemic treatment that statistically reduced the severity of radiation dermatitis in a patient.

Topical drug-induced vasoconstriction reduces epidermal stem cell damage by transiently inducing local skin hypoxia during radiotherapy. By reducing the formation of ROS and directly suppressing “fixation” (which requires O_2_) of ROS damage to DNA, topical vasoconstrictors shift the dose response curve for acute skin radiation damage and reduce the frequency of the grade 2 and 3 radiodermatitis that causes severe patient discomfort and requires clinical intervention and treatment delays. Hall [15] attributes 70 + % of the cell damage from ionizing radiation to “fixed” ROS-mediated DNA damage, which is significantly suppressed by hypoxia.

In a previous Phase I safety study in 18 normal volunteers (unpublished), topically applied NG12-1 elicited a transient skin blanch with the maximum blanch seen 15-20 min post-application. The blanch intensity seen at Dose Level 1 was less pronounced and of shorter duration than that seen at Dose Levels 2–4, which had 2-4-fold increases in drug concentration. For all 18 subjects in the study, the daily skin blanch scores in the sixth week of treatment were no different than the scores in week one, indicating that there was no adrenergic “receptor fatigue” over the 6 week Phase I study.

There is no evidence that the NG12-1 topically applied to skin enters the systemic circulation. Through three clinical studies, where more than 40 subjects received more than 1000 topical NG12-1 applications, there were no detectable occurrences of elevated blood pressure or tachycardia. In a pilot pharmacokinetics study (unpublished), no detectable NG12-1 was found in patient plasma samples following topical applications.

Although protection of target tumor cells is a theoretical risk of any treatment that protects surface skin cells from radiation, constriction of subcutaneous vessels, located ~1 mm below the skin surface, is not likely to present risk to post-lumpectomy radiotherapy patients. First, breast cancer recurrence sites are typically within the breast parenchyma in the region of the primary tumor or at distant metastatic sites, rarely within the 1 mm skin depth that is made hypoxic with this topical vasoconstriction [16, 17]. Second, in human tumor protection studies in a nude mouse xenograft model, topical vasoconstrictors applied onto the skin directly over subcutaneous human tumor xenografts, induced clear blanch of the overlying skin, but conferred zero reduction in radiation-induced killing of three different human tumor cell populations [18].

The limitations of the present pilot Phase IIa study include the small number of treated subjects, the lack of blinding between drug-treated and placebo-treated topical sites, and a 50 cm^2^ SDAS within a much larger chest and axilla radiation field.

## Conclusions

We conclude that thirty, daily, NG12-1 treatments, topically applied minutes before radiotherapy, were well tolerated and they conferred a statistically significant reduction in radiodermatitis severity (*P* = 0.022).

## Abbreviations

COMT: Catechol-O-methyltransferase
ECG: Electrocardiogram
Gy: Gy
IRB: Institutional review board
MAO: Monoamine oxidase
ROS: Reactive oxygen species
SDAS: Study drug application site
